# Associations of the risk of lung cancer with serum 25-hydroxyvitamin D level and dietary vitamin D intake: A dose-response PRISMA meta-analysis

**DOI:** 10.1097/MD.0000000000012282

**Published:** 2018-09-14

**Authors:** Hu Wei, Hu Jing, Qian Wei, Guo Wei, Zhou Heng

**Affiliations:** a Department of Radiology, the Fifth Hospital of Wuhan; b Department of Pathology, Renmin Hospital of Wuhan University, Wuhan, Hubei Province, People's Republic of China.

**Keywords:** 25-hydroxyvitamin D, lung cancer, meta-analysis, vitamin D

## Abstract

The associations of the risk of lung cancer with the vitamin D intake and serum level are controversial. We performed a comprehensive dose-response meta-analysis to evaluate the precise relationships between the above mentioned parameters.

We performed a web search of the PubMed, Medline, and Embase databases to identify potential studies that evaluated the relationships between vitamin D intake or serum 25-hydroxyvitamin D (25([OH]D) levels and the risk of lung cancer on December 5, 2017. According to the inclusion and exclusive criteria, 16 studies were included in this meta-analysis. The pooled relative risks (RRs) with 95% confidence intervals (CIs) were used to assess the strength of the associations. A dose-response analysis was conducted to quantitate the relationship between the serum 25(OH)D or vitamin D intake and the risk of lung cancer.

The pooled RR (highest level vs lowest level) showed that the serum 25(OH)D level was not associated with the risk of lung cancer (RR = 1.046, 95% CI = 0.945–1.159). A high vitamin D intake was inversely correlated with the lung cancer risk (RR = 0.854, 95% CI = 0.741–0.984). No significant dose-response relationship was observed between the serum 25(OH)D level and the lung cancer risk. The linearity model of the dose-response analysis indicated that with every 100 IU/day increase in vitamin D intake, the risk of lung cancer decreased by 2.4% (RR = 0.976, 95% CI = 0.957–0.995, *P* = .018).

A high vitamin D intake provides limited protection against lung cancer carcinogenesis.

## 1 Introduction

Lung cancer has been the most common and lethal cancer worldwide for several decades. In 2018, lung cancer is estimated to account for 11,2350 new cancer cases (13% of the total cancer cases) in the United States.^[1]^ Although treatments for lung cancer are developing rapidly, the overall survival of patients with lung cancer is relatively low (5-year survival rates, 16% in the United States and 10% in the United Kingdom). In 2017, approximately 1.6 million patients died of lung cancer, which is approximately 26% of all cancer-related deaths.^[2,3]^ Hence, early diagnosis and prevention is important to decrease the morbidity and mortality of this disease.

Vitamin D is mainly obtained from 2 pathways in humans: synthesis in the skin by exposure to ultraviolet radiation from sunlight and direct intake from dietary food. Vitamin D is hydroxylated to the circulating form—25-hydroxyvitamin D (25[OH]D)—in the liver and transformed into 1,25-hydroxyvitamin D(1,25[OH]D) in the kidney. In addition, 25(OH)D has a longer half-life than 1,25(OH)D and is considered an appropriate reflector of the vitamin D levels in serum.^[4]^

Vitamin D takes part in many cell functions including cell apoptosis, differentiation, metastasis, angiogenesis, and proliferation.^[5–7]^ Previous studies have reported that the vitamin D level is associated with a decreased risk of different cancers including breast,^[8]^ colorectal,^[9]^ and kidney^[10]^ cancers. However, no association between the vitamin D level and prostate,^[11]^ esophageal,^[12]^ pancreatic,^[13]^ skin,^[14]^ and gastric^[15]^ cancers was reported. Previous meta-analyses have reported an association between high serum 25-hydroxyvitamin D levels and a reduced risk of lung cancer.^[16,17]^ However, the dose-response relationship between the risk of lung cancer and serum 25(OH)D levels or dietary vitamin intake is unclear. Therefore, this comprehensive dose-response meta-analysis aimed to evaluate the dose-response relationship of the risk of lung cancer with the vitamin D intake and serum 25(OH)D level.

## 2 Materials and methods

This meta-analysis was performed according to the latest Preferred Reporting Items for Systematic Reviews and Meta-analyses (PRISMA).^[18,19]^

### 2.1 Literature search

A systematical search of the PubMed, Medline, and Embase databases was performed up to December 5, 2017, by 2 reviewers (HW and HJ) using the following search terms: vitamin D or 25-hydroxyvitamin D or 25 hydroxyvitamin D or 25(OH)D) AND lung AND (cancer or carcinoma or adenocarcinoma or squamous carcinoma or tumor or non-small cell lung cancer or small cell lung cancer or NSCLC or SCLC). In addition, the reference lists of the original articles were reviewed, from which other available publications were selected manually. No language restrictions were imposed in the process of searching.

### 2.2 Study selection

The inclusion criteria for the studies were as follows: a case-control or cohort design; reports on the associations of the risk of histologically diagnosed lung cancer with the serum 25(OH)D levels and dietary vitamin D intake; inclusion of relative risk (RR), hazard ration (HR), odds ratio (OR) with 95% confidence interval (CI), or associated data to estimate the association of the risk of lung cancer with the highest versus lowest vitamin D levels; and indication of the number of cases and participants and eligible dose concentration for dose-response analysis. The following studies were excluded from the analysis: studies that did not evaluate the associations between the vitamin D intake and lung cancer risk, and studies that used the serum 1,25(OH)D level as an indicator of the vitamin D level. In the event of duplicate publications, the most complete or most recent publication was used.

### 2.3 Data extraction and quality assessment

Data were extracted by 2 independent researchers (HW and QW). The following information were selected according to the criteria listed previously: publication year, the first author's name, country, study design, sample size, vitamin D, or serum 25(OH)D level, measurement method, adjusted variables, risk estimates and 95% CI for evaluating the highest vitamin D levels versus lowest vitamin D levels. We choose the maximally adjusted rations as the only evaluation index for preventing potential confounders when studies reported several multivariable adjusted-effect estimates. When studies did not set groups of lowest dose concentration as reference groups, the EXCEL macro document (RRest9) was used for the reference group transforming, and data was re-calculated according to the instructions.^[20]^ All controversial questions were resolved by asking the third author (GW).

The New Castle–Ottawa quality assessment scale (NOS) system, which has been validated as a comprehensive tool for assessing the quality of observational studies in meta-analysis, were used to assessed study quality.^[21]^ NOS evaluating details including the following 3 aspects awarded a total 9 items: selection of participants and measurement of exposure (4 items), comparability (2 items), and evaluation of methodological quality outcome (3 items). Studies with 7 score or higher score were considered as high quality studies.^[22,23]^

### 2.4 Statistical analysis

Pooled risk estimates (RR or OR) with 95% CI were used to identify associations between the risk of lung cancer and the vitamin D intake or serum 25(OH)D level. The heterogeneity was evaluated with the *I*^*2*^ statistic. Cut-off values for *I*^*2*^ were set at 25%, 50%, and 75% for low, moderate, and high degrees of heterogeneity, respectively. When the heterogeneity was <25%, a fixed-effect model was chosen; otherwise, a random-effect model was chosen.^[24]^ When the heterogeneity was significant, sensitivity analysis was performed to assess the robustness of the pooled results by excluding one study at a time. Publication bias was assessed using the Begg rank model and Egger linear model.^[25]^ Subgroup analysis was performed according to the country, mean age, study design, smoking, gender, baseline 25 (OH)D levels, measurement method, pathological type, and study quality. We performed a dose-response meta-analysis by using the correlated natural logs of the RRs or ORs with their standard error across all vitamin D-intake categories. To drive the dose-response curve, the restricted cubic splines with 4 knots at the 5%, 35%, 65%, and 95% percentiles of distribution were used in order to evaluate the potential curvilinear relations.^[26]^ All statistical analyses were performed using Stata 12.0 software (StataCorp LP, College Station, TX).

This study does not include experiments with animals or humans. Due to the nature of the study, ethical consent was not required.

## 3 Results

### 3.1 Summary of the study characteristics

**Figure 1:**
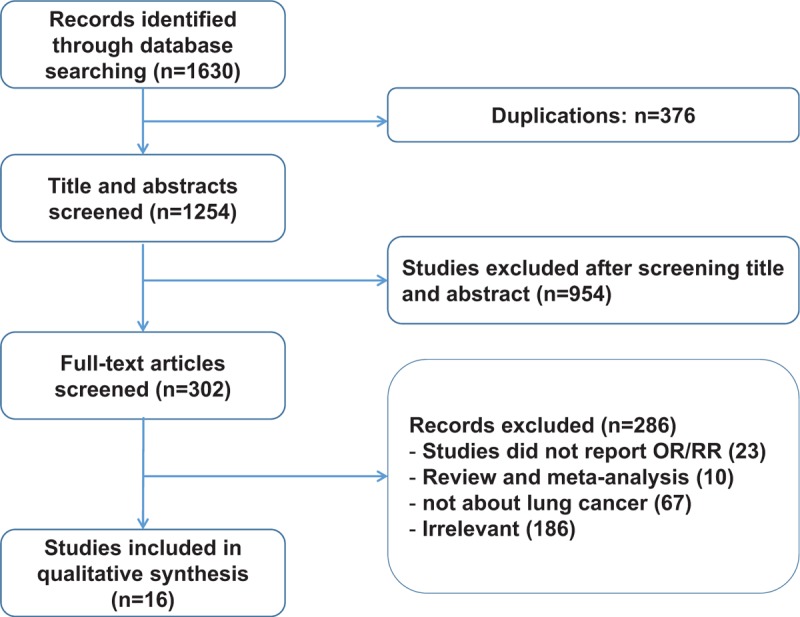
The flow diagram of the literature search, analysis, and exclusion criteria used in the meta-analysis.

After screening the titles and abstracts of 1254 articles, which were identified from the initial search of online databases, 952 studies were excluded. The eligibility of the remaining 302 studies was assessed by full-text reading. Finally, 16 studies were included in our meta-analysis. The search results and eligible literature selection process are showed in Figure 1.

The 16 studies selected were published between 2006 and 2017 and included 7823 lung cancer patients and 27,2304 control subjects.^[27–42]^ Three studies were conducted in China,^[27,30,39]^ 5 studies were conducted in the United States,^[29,35,36,38,42]^ 7 studies were performed in Europe,^[28,31,33,34,37,40,41]^ and 1 study was conducted in Australia.^[32]^ Seven studies reported a mean age of <60 years among participants,^[30,34–39]^ and 8 studies reported a mean age of >60 years.^[27–29,31,32,40,42]^ Eleven studies were cohort studies,^[27,31–33,35,37–39,41,42]^ and 5 studies were case-control studies.^[28–30,36,40]^ Five studies included individuals who smoked,^[30,35,36,41,42]^ and 4 studies included non-smokers.^[29,30,39,41]^ In terms of the pathological type, 5 studies reported on non-small cell lung cancer (NSCLC),^[27,29,30,41,42]^ 3 studies reported on small cell lung cancer (SCLC),^[27,41,42]^ 4 studies reported on adenocarcinoma,^[27,29,41,42]^ and 3 studies reported on squamous carcinoma.^[27,41,42]^ In addition, 5 studies investigated the association between the vitamin D level and the risk of lung cancer in women,^[29,37,39–41]^ and 3 studies evaluated this association in men.^[32,36,37]^ Eight studies considered the mean baseline of 25(OH)D level as >50 nM,^[27,28,31–33,35]^ and 4 studies considered this level as <50 nM.^[29–31,34,36,37]^ Nine studies detected serum 25(OH)D concentrations using the chemiluminescent immunoassay (CIA) method,^[27–29,31–34,36]^ and 3 studies used the radioimmunoassay (RIA) method.^[30,35,37]^ Eleven studies matched the high score with the NOS scale,^[27,29,31–33,36,37,39–42]^ and the remaining studies had a low NOS score.^[28,30,34,35,38]^ Eight studies were included in the dose-analysis of the serum 25(OH)D level and lung cancer risk,^[27–29,31,32,35–37]^ and 3 studies were included in the dose-analysis of the vitamin D intake and lung cancer risk.^[39,41,42]^ The main profiles of the included 16 included articles were summarized in Table 1.^[27–42]^

**Table 1:**
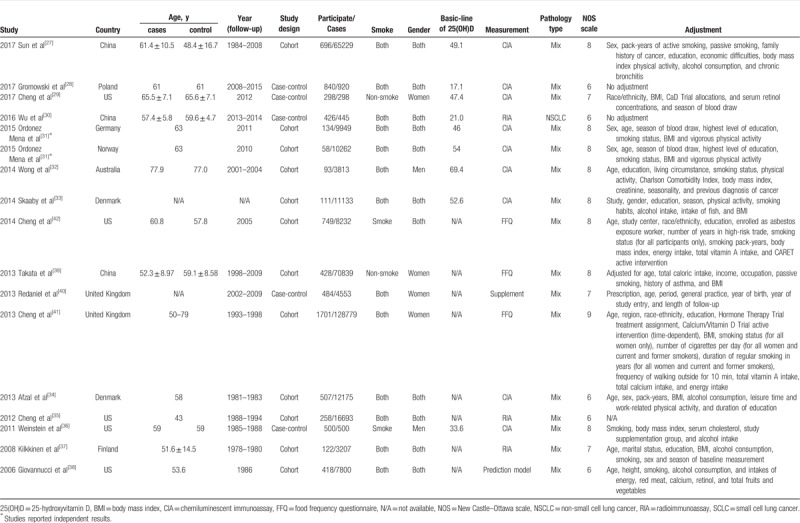
Characteristics of studies included in the meta-analysis.

### 3.2 Serum 25(OH)D level and risk of lung cancer

**Figure 2:**
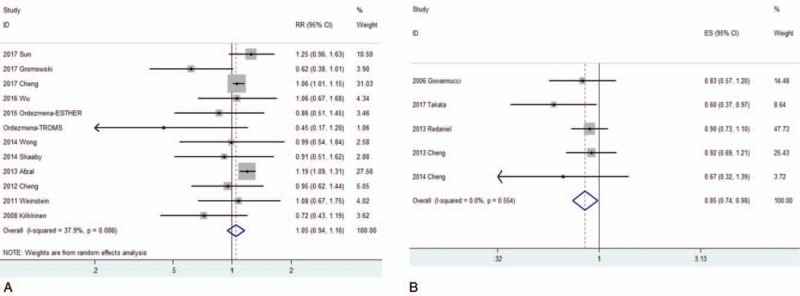
Forest plots of the association between the 25-hydroxyvitamin D (A) or dietary vitamin D intake (B) and the risk of lung cancer.

To evaluate the link between the serum 25(OH)D level and lung cancer risk, totally 4 case-control studies^[28–30,36]^ and 8 cohort studies^[27,31–33,35,37]^ including 4043 patients and 13,4624 controls were analyzed. Due to the significant heterogeneity (*P* = .038, *I*^*2*^ = 50.9%) indicated that a random-effect model was applied. The pooled RR for the highest level versus the lowest level was 1.046 (95% CI = 0.945–1.159, Table 2, Fig. 2A), which suggested no significant association between serum 25(OH)D level and the risk of lung cancer.

**Table 2:**
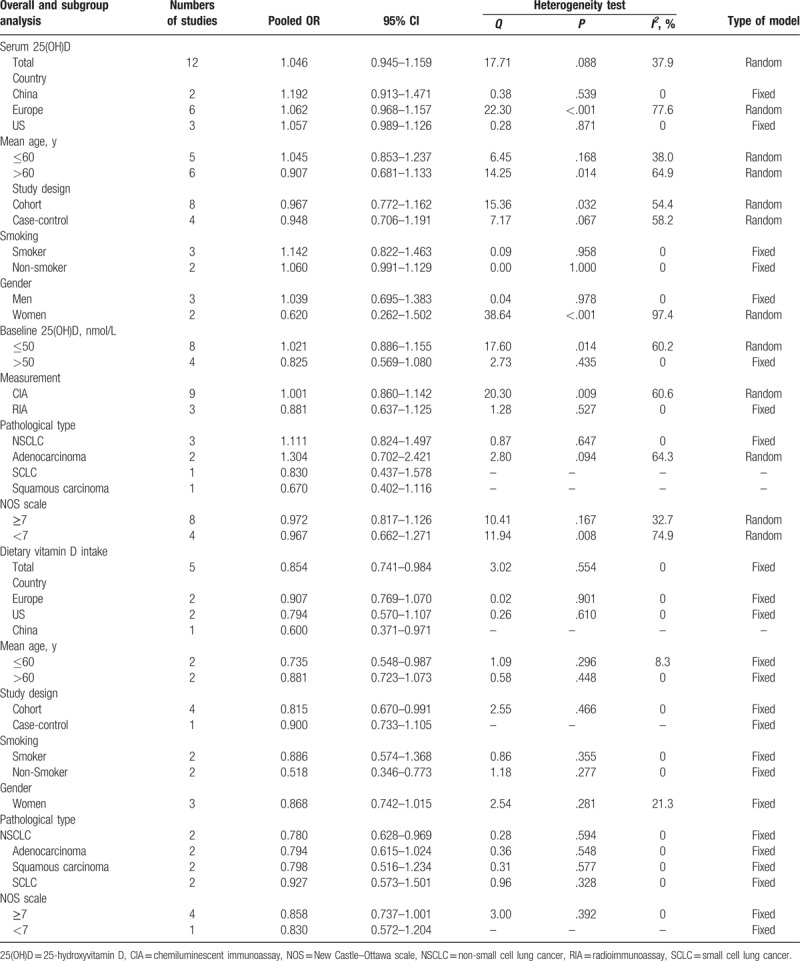
Results including overall and subgroup analysis of pooled OR, 95% CI, heterogeneity test, and publication bias.

Table 2 shows the detailed results of the specific stratified analysis based on country, mean age, study design, smoking status, gender, baseline of 25(OH)D levels, measurement method, pathological type, and NOS quality. Subgroup analysis of women (RR = 0.620, 95% CI = 0.262–1.502), baseline 25(OH)D level >50 nM (RR = 0.825, 95% CI = 0.569–1.080), SCLC (RR = 0.830, 95% CI = 0.437–1.578), and squamous carcinoma (RR = 0.670, 95% CI = 0.402–1.116) suggested an inverse relationship between the serum 25(OH)D level and the lung cancer risk. In contrast, all other stratified analysis suggested no association between serum 25(OH)D level and lung cancer risk.

**Figure 3:**
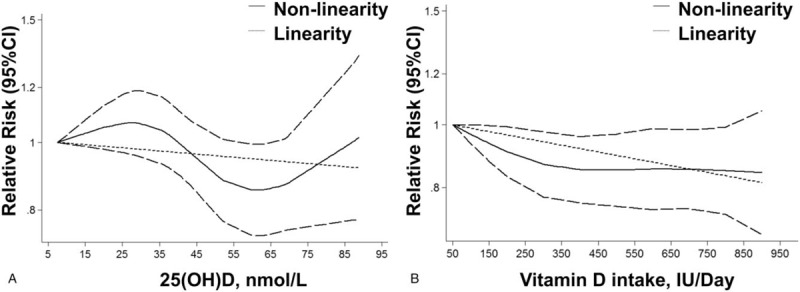
Linearity and non-linearity dose-response relationships between the risk of lung cancer and the 25-hydroxyvitamin D (A) and dietary vitamin D intake (B).

To determine the relationship between the serum 25(OH)D level and lung cancer risk, a dose-response analysis including 5 cohort studies and 4 case-control studies was performed. As shown in Figure 3A, the linearity (*P* = .349) or non-linearity tests (*P* = .14) of the dose-response analysis suggested no association between the serum 25(OH)D level and the risk of lung cancer.

### 3.3 Dietary vitamin D intake and risk of lung cancer

To evaluate the relationship between the dietary vitamin D intake and the risk of lung cancer, one case-control study^[40]^ and 4 cohort studies^[38,39,41,42]^ including 3780 patients and 13,7680 controls were analyzed. Since no significant heterogeneity (*P* = .038, *I*^*2*^ = 50.9%), we used a fixed effect model. The multivariable-adjusted RR of lung cancer for the highest level versus the lowest level of dietary vitamin D intake was 0.854 (95% CI = 0.741–0.984, Table 2, Fig. 2B), which suggested that an association between an increased dietary vitamin D intake and a small reduction in the risk of lung cancer.

The results of the subgroup analyses according to country, mean age, study design, smoking status, gender, pathological type, and NOS quality were similar as those of the comprehensive meta-analysis. Table 2 shows the detailed results of the stratified analysis.

The dose-response analysis including 3 cohort studies suggested that a 100 IU/day increase in the vitamin D intake decreased the risk of lung cancer by 2.4% degree (RR = 0.976, 95% CI = 0.957–0.995, *P* = .018, Fig. 3B). In addition, the non-linearity test also indicated a weak positive association between the vitamin D intake and lung cancer risk (*P* = .045).

### 3.4 Sensitivity analysis and publication bias

When the heterogeneity was high, we used the sensitivity analysis was necessary. Sensitivity analysis of the serum 25(OH)D level was performed by omitting one included study at a time and showed stable results in the meta-analysis. Publication bias was evaluated by the Begg test and Egger test. In the analysis of the serum 25(OH)D and the risk of lung cancer, the *P* values for the Begg test and Egger test were 0.244 and 0.07, respectively. In the analysis of vitamin D intake and risk of lung cancer, the *P* values for the Begg test and Egger test were 0.100 and 0.09, respectively. No significant publication bias was detected in the meta-analysis.

## 4 Discussion

Our meta-analysis indicated that the serum 25(OH)D level was not associated with risk of lung cancer. In addition, the dose-response analysis also showed no association between the serum 25(OH)D level and the risk of lung cancer. The comprehensive meta-analysis, dose-response analysis, and subgroup analysis revealed that an increase in the vitamin D intake was associated with a decrease in the risk of lung cancer. Moreover, the serum 25(OH)D level was not associated with risk of lung cancer in most subgroup analyses except for women, baseline 25(OH)D level >50 nM, SCLC, and squamous carcinoma.

Studies have reported that an increase in the vitamin D level is associated with a decrease in the risk of different cancers including breast,^[8]^ colorectal,^[9]^ and kidney,^[10]^ cancers. Mechanistically, vitamin D contributes to the transcription level of cathelicidin antimicrobial peptide genes and the translation of CD14, a co-receptor for identifying bacterial lipopolysaccharides, both of which are important for innate immunity in the lung, and improve host defense.^[5]^ Several cell and animal experiments have found that the active metabolite of 25(OH)D—1,25(OH)_2_D suppresses angiogenesis and cancer cell growth by inhibiting the response to vascular endothelial growth factor.^[6]^ In addition, 1,25(OH)_2_D inhibits metastasis and proliferation of lung cancer by prevents other signal pathways including mutations of *K-ras* and epidermal growth factor receptor and the Wnt/β-catenin pathway.^[43–45]^ In addition, 1,25(OH)_2_D also increased the expression of E-cadherin, a glycoprotein that is vital for cell adhesion, and prevented cancer cell metastases.^[46]^ Importantly, 1,25(OH)_2_D decreased the expression of cyclooxygenase-2 and prostaglandin, and contributed to preventing cancer cell growth and angiogenesis.^[47]^ Although vitamin D theoretically inhibits cancer cell angiogenesis and proliferation, and promotes cancer cell apoptosis, differentiation and metastasis theoretically,^[5–7]^ it does not stop some cancers occurring including prostate, esophageal, pancreatic, skin, and gastric cancers.^[11–15]^

Although previous meta-analysis suggested that a high concentration of serum 25(OH)D level protects against lung cancer occurring,^[16,17]^ our meta-analysis included more case-control and cohort studies and suggested no relationship between the serum 25(OH)D level and lung cancer risk. In addition, our dose-response meta-analysis including both linearity and non-linearity tests all confirmed the comprehensive results of our meta-analysis. The results of the non-linearity analysis suggested that the risk of lung cancer decreased when the 25(OH)D concentration was low, up till 60 nM (RR = 0.85), after which the risk increased. Interestingly, our results were consistent with those of a previous dose-response meta-analysis that included only cohort studies,^[17]^ which suggested that a high vitamin D intake cannot provide more protection against lung cancer. Feng et al^[48]^ have found that a high serum 25(OH)D level was not associated with the overall survival of lung cancer. In the subgroup analysis, most results were consistent with those of the comprehensive meta-analysis except the results of the subgroup analysis of women, baseline 25(OH)D levels >50 nM, SCLC, and squamous carcinoma.

In the analysis of dietary vitamin D intake and lung cancer risk, we found that the comprehensive results were similar to those of the non-linearity test of dose-response analysis of the serum 25(OH)D levels, which suggested that a high level of vitamin D intake significantly decreases the risk of lung cancer. Therefore, we performed a dose-response meta-analysis of the dietary vitamin D intake and lung cancer risk, which suggested that both results of both the non-linearity and linearity tests were significant. However, the non-linearity tests suggested that when the dietary vitamin D intake exceeded 400 IU (RR = 0.85), it provided limited protection against lung cancer occurring. However, because diet comprises only a portion of the total vitamin D intake, these results may not be conclusive.^[4]^

Our meta-analysis had a few limitations. First, the number of included studies in some subgroup analysis was small, which may make influence on the last conclusions. Second, the original studies did not provide individual data, and the results of our meta-analysis were evaluated by pooled RR and the associated 95% CI, which prevented further detailed analysis and precise results. Hence, our results should be interpreted with caution.

In conclusion, our comprehensive meta-analysis indicated no association between a high level of circulating 25(OH)D in serum and the risk of lung cancer. The dose-response analysis of the dietary vitamin D intake indicates that every 100 IU/day intake of vitamin D accounts for a 2.4% decrease in the risk of lung cancer. Therefore, our results suggest that a high level of vitamin D intake provides the limited protection by decreasing the risk of lung cancer. Furthermore, the present meta-analysis suggested that well-designed, large-scale, observational, prospective studies should be conducted in the future to validate the precise relationship between the vitamin D intake, and lung cancer risk.

## Author contributions

**Conceptualization:** Guo Wei.

**Data curation:** Hu Jing, Guo Wei.

**Formal analysis:** Guo Wei.

**Funding acquisition:** Guo Wei.

**Investigation:** Hu Jing.

**Methodology:** Qian Wei, Guo Wei.

**Software:** Hu Wei.

**Validation:** Zhou Heng.

**Writing – original draft:** Hu Wei.

**Writing – review & editing:** Guo Wei, Zhou Heng.
